# The Postpartum Specific Anxiety Scale: Confirmatory factor analyses and relationships with birth experience

**DOI:** 10.1007/s00737-022-01233-9

**Published:** 2022-04-30

**Authors:** Victoria Fallon, Siân M. Davies, Paul Christiansen, Joanne A. Harrold, Sergio A. Silverio

**Affiliations:** 1 Department of Psychology, Institute of Population Health, Faculty of Health and Life Sciences, University of Liverpool, Liverpool, UK; 2 School of Psychology, Faculty of Health, Liverpool John Moore’s University, Liverpool, UK; 3 Department of Women & Children’s Health, School of Life Course & Population Sciences, Faculty of Life Sciences & Medicine, King’s College London, 10th Floor North Wing, St. Thomas’ Hospital, Westminster Bridge Road, Lambeth, London, SE1 7EH UK

**Keywords:** Maternal anxiety, Birth experience, Psychometrics, Birth satisfaction, Child-bearing specific measures of mood

## Abstract

The Postpartum Specific Anxiety Scale [PSAS] was developed and validated as a research tool with a four-factor structure; with predictive validity corroborated in studies examining infant-feeding and maternal bonding outcomes. The PSAS has not been examined in relation to birth experiences. We aimed to confirm the PSAS four-factor structure and examine these domains of anxiety in relation to subjective and objective birth experiences. Postpartum mothers (≤ 12-months; *N* = 500) completed the PSAS alongside measures of subjective birth satisfaction and objective obstetric interventions/complications. Confirmatory factor analyses [CFA] tested eight models, theoretically derived from the preceding exploratory work. Structural equation modelling [SEM] tested associations between each PSAS factor and birth experience variables in the best-fitting model. An identical 51-item four-factor model fits the data well. SEM analyses revealed associations between lower perceptions of quality of intrapartum care and increased maternal competence and attachment anxieties, practical infant care anxieties, and infant safety and welfare anxieties. High subjective stress and negative emotional response to labour were associated with increased psychosocial adjustment to motherhood anxieties. Specific associations were found between neonatal care unit admission and practical infant care anxieties; and infant asphyxia and infant safety and welfare anxieties. Findings confirm construct and convergent validity of the four-factor PSAS and its use in measuring postpartum anxiety. Unique associations were also identified, indicating specific subjective and objective experiences occurring during birth may elicit a differential anxiety response, in that they are related to specific forms of postpartum anxiety which occur during the first postpartum year.

## Introduction

The increasing recognition and prevalence of postpartum anxiety along with the persistent and far-reaching effects for mothers and infants poses a significant health and economic burden for healthcare systems and wider society (Bauer et al. 2014; Field 2018). Until recently, in research studies and clinical practice, postpartum anxiety was identified and measured using general tools which were not designed or validated for use in postpartum populations (e.g. The State-Trait Anxiety Inventory [STAI]; Spielberger et al. 1983; and the Generalised Anxiety Disorder-7 [GAD-7]; Spitzer et al. 2006]). This was evidenced in a systematic review of anxiety measures validated in perinatal populations, which reported that no factor analyses had been conducted on either the STAI or the GAD in a perinatal sample (Meades and Ayers 2011). The use of general measures of anxiety within childbearing populations has been increasingly recognised as problematic (National Collaborating Centre for Mental Health UK 2018). For instance, items such as ‘I feel rested’ may not be applicable to mothers as disrupted sleep is a normal aspect of motherhood during the postpartum (Galland et al. 2012) and therefore may artificially inflate anxiety scores (Matthey et al. 2003). Conversely, general items fail to capture various types of maternal and infant focused concerns and therefore low scores on these measures do not necessarily imply the absence of postpartum anxiety (Phillips et al. 2009).

The Postpartum Specific Anxiety Scale (PSAS; Fallon et al. 2016) is a 51-item questionnaire designed to measure the frequency of maternal and infant focused anxieties experienced in the last week. The scale was originally developed from qualitative interviews with postpartum mothers in the UK. It was reviewed and refined by an expert panel, underwent pre-testing, and demonstrated excellent acceptability and comprehensibility in a postpartum population. Initial validity and reliability testing in a sample of mothers of infants up to six months of age in English-speaking countries (*n* = 800) demonstrated the PSAS had good construct and convergent validity and excellent internal and test–retest reliability. The scale had a simple four-factor structure expressed as: (1) maternal competence and attachment anxieties, (2) infant safety and welfare anxieties, (3) practical infant care anxieties, and (4) psychosocial adjustment to motherhood.

Since the initial publication in 2016, a 16-item research short-form PSAS has been developed and validated in three random samples containing over 2000 postpartum women with infants aged from birth to twelve months (PSAS-RSF; Davies et al. 2021). A rapid-response 12-item version for use in the COVID-19 pandemic has also been developed and validated from a sample of postpartum women in the first UK lockdown (PSAS-RSF-C; Silverio et al. 2021). Both versions demonstrated robust psychometric properties and a retained an identical four-factor structure demonstrating factor stability, validity, and reliability across different samples of perinatal populations and contexts. Validation of any measure is an iterative process, and the authors of the original PSAS have yet to confirm the four-factor structure of the 51-item scale in another English-speaking sample. It is also necessary to examine the psychometric potential of the measure across the first postpartum year to increase the accessibility of the measure for researchers.

The predictive validity of the PSAS has also been examined and confirmed in relation to both infant-feeding outcomes and perceptions of feeding behaviours (Fallon et al. 2018) and maternal-infant bonding behaviours (Fallon et al. 2021). It was identified in both of these papers that the PSAS predicted unique variance in these maternal and infant outcomes after controlling for a general measure of anxiety (i.e. STAI; Spielberger et al. 1983). However, no work has examined correlates of the individual PSAS subscales. This may provide a greater level of detail in terms of identifying specific risk factors and mechanisms of postpartum anxiety and offers opportunity for more targeted intervention of anxiety symptoms.

Childbirth is a life-changing transitional event for women which can influence the relationship between mother and infant, maternal self-efficacy, and parenting confidence, with risk factors for psychopathology. Maternal and infant focused anxiety is centred around these domains of parenting and therefore may be exacerbated by a difficult birth, which may physically and emotionally impede early mother–infant attachment (Reisz et al. 2019). Birth experience refers to both objective interventions and complications occurring during labour and birth and the subjective experience of labour and birth as perceived by the individual woman (Baptie 2021). Reviews which assess both objective and subjective factors of labour and birth suggest subjective experience is a better predictor of postnatal trauma compared to objective obstetric interventions or complications (Andersen et al. 2012; Ayers et al. 2016; Dekel et al. 2017; Grekin and O'Hara 2014; Simpson and Catling 2016), but this has yet to be explored as comprehensively in other domains of mental health after birth. While there is some evidence linking postpartum depression with childbirth experiences (Bell and Andersson 2016), a recent review suggests some childbirth experiences may be associated with anxiety (Field 2018). These include objective experiences such as caesarean delivery and subjective experiences such as fear of childbirth, lack of control, and less self-confidence (Field 2018). However, work in this area is sparse and has—to date—solely used general measures of anxiety.

### Aims

Using the original 51-item PSAS (Fallon et al. 2016), we address the following aims to:

- Confirm the four-factor structure of the PSAS using confirmatory factor analyses in an English-speaking sample of mothers with infants up to 12 months of age.
- Model associations between subjective and objective birth experiences and the individual PSAS subscales, hypothesising that low birth satisfaction and obstetric interventions or complications during labour would be associated with higher levels of postpartum specific anxieties in the first year after birth.

## Methods

### Ethics

Ethical approval was sought and granted by the Research Ethics Committee at the University of Liverpool (ref: IPHS/3647).

### Participants

An opportunity sample of English-speaking mothers (*N* = 500) with infants aged between birth and 12 months were recruited via advertisements posted on Facebook, Twitter, and other parenting sites and social media platforms which contained a link to an on-line survey about anxiety and birth experiences. Mothers had to be ≥ 18-years to participate. Maternal age ranged between 19 and 44 years (*M*_*Ag*e_ = 33.41 years, SD = 5.21) and infant age ranged between birth and 12 months (*M*_*Age*_ = 25.30 weeks, SD = 14.86). Women were predominately white (95%), from the UK (75%), married (58%), university educated (65%), and professionals (39%). Furthermore, 123 had a self-reported clinical diagnosis of anxiety (25%) and 90 had a self-reported clinical diagnosis of depression (18%). In relation to objective birth experience, 188 women had labour induced (38%), 123 women underwent a Caesarean section (25%), 65 women reported augmentation of labour (13%), 46 women experienced perineal laceration (9%), 43 women experienced breech birth (9%), 61 women experienced infant admission to neonatal care (12%), 52 women experienced a postpartum infection (7%), 49 women experienced postpartum haemorrhage (10%), 30 women experienced infant umbilical cord issues during birth (5%), 10 women’s infants experienced birth asphyxia (1.5%), and 10 women experienced placenta praevia (1.5%). Mean score for stress and emotional response to childbirth (4-items; minimum score = 0, maximum score = 8) were 4.23 (SD = 1.91), and that for quality of care experienced during childbirth (2-items; minimum score = 0, maximum score = 4) were 0.70 (SD = 1.26). Maternal and infant demographic and birth experience information can be found in Tables 1 and 2.

**Table 1:** Maternal and infant characteristics (*N* = 500)

Maternal characteristic	Value	Infant characteristic	Value
Maternal age (mean years ± SD)	33.41 (5.21)	Infant age (mean weeks ± SD)	25.30 (14.86)
Country of Residence (N/%)		Infant birth weight (mean weeks ± SD)	
UK	377 (75.4)	Birth order (N/%)	
Ireland	9 (1.8)	1^st^	297 ()
USA and Canada	87 (17.4)	2^nd^	132 ()
Australia & NZ	2 (0.4)	3^rd^	55 ()
Other European	22 (4.4)	4^th^	10 ()
Other Non-European	3 (0.6)	5^th^ or more	4 ()
Ethnicity (N/%)		Timing of birth (N/%)	
White	474 (94.8)	Premature (< 37 weeks)	52 (11.8)
Pakistani	1 (0.2)	Early term (> 37 < 39 weeks)	118 (23.6)
Black Caribbean	1 (0.2)	Full term (39 weeks)	126 (25.2)
Chinese	3 (0.6)	Post-term (> 40 weeks)	197 (39.4)
Indian	3 (0.6)	Multiple birth (N/%)	
Other	17 (3.4)	Yes	11 (2.2)
Prefer not to say	1 (0.2)	No	489 (97.8)
Marital Status (N/%)		Current feeding method (N/%)	
Married	292 (58.4)	Exclusively breastfeeding (100%)	228 (45.6)
Co-habiting	193 (38.6)	Predominantly breastmilk (over 80%) with a little formula milk (20%)	46 (9.2)
Separated	2 (0.4)	Mainly breastmilk (50–80%) with some formula milk	10 (2.0)
Single	13 (2.6)	A combination of both breastmilk (50%) and formula milk (50%)	14 (2.8)
Occupation (N/%)		Mainly formula milk (50–80%) with some breastmilk	10 (2.0)
Managers, directors, senior officials	48 (9.6)	Predominantly formula milk (over 80%) with a little breastmilk (20%)	10 (2.0)
Professionals	195 (39.0)	Exclusively formula feeding (100%)	182 (36.4)
Associate professionals and technical	14 (2.8)		
Administrative and secretarial	54 (10.8)	Maternal Anxiety	Mean (± SD)
Skilled trade	15 (3.0)	Overall PSAS	113.58 (25.21)
Caring, leisure and other service	61 (12.2)	PSAS Factor 1	28.89 (8.71)
Sales and customer service	61 (12.2)	PSAS Factor 2	24.62 (6.69)
Process, plant and machine Operatives	1 (0.2)	PSAS Factor 3	15.04 (4.36)
Elementary	12 (2.4)	PSAS Factor 4	
Not in paid occupation	39 (7.8)		
Education attainment (N/%)			
Postgraduate education	120 (24.0)		
Undergraduate education	207 (41.4)		
A-Levels or college equivalent	108 (21.6)		
GCSEs or secondary school equivalent	40 (8.0)		
No qualifications	8 (1.6)		
Other qualification	17 (3.4)		
Current diagnosis of anxiety (N/%)			
Yes	123 (24.6)		
No	370 (74.0)		
Prefer not to say	7 (1.4)		
Current diagnosis of depression (N/%)			
Yes	90 (18.0)		
No	408 (81.6)		
Prefer not to say	2 (0.4)

**Table 2:** Birth experience characteristic (*N* = 500)

Subjective birth experience	Value
Overall BSS-RI (mean ± SD)	4.93 (1.88)
Overall BSS-RI-SE (mean ± SD)	4.23 (1.91)
Overall BSS-RI-QC (mean ± SD)	0.70 (1.26)
Objective birth experience characteristic	Value
Caesarean section (N/%)	
Yes	123 (24.6)
No	377 (75.4)
Induction (N/%)	
Yes	188 (37.6)
No	312 (62.4)
Augmentation (N/%)	
Yes	65 (13.0)
No	435 (87.0)
Breach (N/%)	
Yes	43 (8.6)
No	457 (91.4)
Neonatal care (N/%)	
Yes	61 (12.2)
No	439 (87.8)
Forceps ventouse (N/%)	
Yes	78 (15.6)
No	422 (84.4)
Infection (N/%)	
Yes	35 (7.0)
No	465 (93.0)
Postpartum haemorrhage (N/%)	
Yes	49 (9.8)
No	451 (90.2)
Perineal laceration (N/%)	
Yes	46 (9.2)
No	454 (90.8)
Setting (N/%)	
Yes	458 (91.6)
No	42 (8.4)
Gestation large (N/%)	
Yes	
No	
Labour length (N/%)	
0–10 h	307 (61.4)
11–20 h	87 (17.4)
21–30 h	52 (10.4)
31–40 h	31 (6.2)
41–50 h	8 (1.6)
51–60 h	3 (0.6)
61–70 h	2 (0.4)
71–80 h	3 (0.6)
91–100 h	7 (1.4)

### Design and procedure

A cross-sectional online survey design was used. Prior to the main survey, participants were asked questions relating to inclusion criteria, with mothers who met the relevant conditions (e.g. maternal age, infant age) being admitted to the main survey. Following this, an electronic information sheet and consent form were provided to confirm informed consent. The survey contained 82-items in total took 15-20 minutes to complete. Every item was compulsory (i.e. ‘forced response’). Upon completion, participants were provided with a full electronic debrief with signposting to relevant support information and were entered into a £25 prize draw. Responses were provided with a unique ID embedded within the survey software programme ensuring anonymity. Additionally, a ‘prevent ballot box stuffing’ option was embedded in the Qualtrics XM survey software preventing repeat responses.

### Measures

#### Demographic questions

Maternal-related demographic questions were asked at the beginning of each survey including maternal age, country of residence, ethnicity, marital status, occupation, educational attainment, self-reported clinical diagnosis of anxiety, and self-reported clinical diagnosis of depression. Infant-related demographic questions were asked including, infant age, birth weight, multiple birth, birth order, and current feeding practices.

#### Anxiety measure: Postpartum specific anxiety scale (PSAS; Fallon et al. 2016)

The PSAS is a 51-item scale examining frequency of maternal and infant focused anxieties experienced by women during the first year following birth. The measure assesses four components of anxiety, specific to the postpartum period. Factor 1 (Maternal Competence and Attachment Anxieties) contains 15-items addressing anxieties relating to maternal self-efficacy, parenting competence, and the mother–infant relationship. Factor 2 (Infant Safety and Welfare Anxieties) contains 11-items relating to fears about infant illnesses, accidents, and cot death. Factor 3 (Practical Infant Care Anxieties) contains 7 items addressing anxieties relating to infant care such as feeding, sleeping, and general routine. Finally, Factor 4 (Psychosocial Adjustment to Motherhood) contains 18 items addressing adjustment concerns following birth of the infant regarding management of personal appearance, relationships and support, work, finances, and sleep. Each answer is given a score on a Likert scale between 1 (‘Not At All’) and 4 (‘Almost Always’), with the maximum score being 204. Furthermore, scores of ≥112 may be indicative of clinically significant anxiety. The PSAS demonstrates excellent reliability (McDonald’s ω = . 95).

#### Objective birth experiences

Objective birth experience questions were co-created with a midwife and addressed the presence of experiences including Caesarean section, induction, augmentation, infant admission to neonatal intensive care unit [NICU], forceps or ventouse assisted birth, postpartum haemorrhage, breech birth, postpartum maternal infection, infant asphyxia, placenta praevia, umbilical cord issues, and perineal laceration. These were presented as a list of different experiences and asked participants to tick which ones applied to their birth.

#### Subjective birth experiences: Birth satisfaction scale—revised indicator (BSS-RI; Martin et al. 2017)

The BSS-RI is a six-item questionnaire measuring women’s satisfaction with care and the experience of labour and birth. The scale assesses two domains of birth satisfaction, stress and emotional response experienced during childbearing and quality of care. Each item is scored on a Likert scale between 0 (‘Agree’) and 2 (‘Disagree’), with higher scores indicating a more positive appraisal of birth satisfaction. The BSS-RI demonstrates good reliability (McDonald’s ω = . 83).

### Method of analysis

#### Confirmatory factor analysis (*N* = 500)

A confirmatory factor analysis was performed using R version 4.0 using diagonally weighted least squares estimation (Mîndrilã 2010). Items were free to load onto their corresponding latent factors, and latent factors were free to correlate with one another. Model fit was assessed using the Comparative Fit Index [CFI] and the Tucker–Lewis Index [TLI], with values of above .90 being deemed acceptable and values of .95 being deemed good (Hu and Bentler 1999); the root mean square error of approximation [RMSEA] where values of .05 and under are deemed good, values of .08 and under are deemed fair, values between .08 and .10 are deemed mediocre, and values over .10 are deemed a poor fit (MacCallum et al. 1996); and the standardised root mean square residual [SRMR], where values less than .08 are considered a good fit (Hu and Bentler 1999). Additionally, modification indices were inspected, covariance pathways were added between error terms (if >20 and providing they were conceptually appropriate, and the items loaded onto the same factor).

#### Structural equation modelling

Linear regressions were then conducted using diagonally weighted least squares estimations to test associations between the subjective birth experience subscales (stress and emotional response to childbirth; quality of care experienced during childbirth) and the PSAS subscales. Binomial logistic regressions were then conducted using generalised linear model estimations to test associations between objective birth experiences and the PSAS subscales.

## Results

### Confirmatory factor analyses

**Fig. 1:**
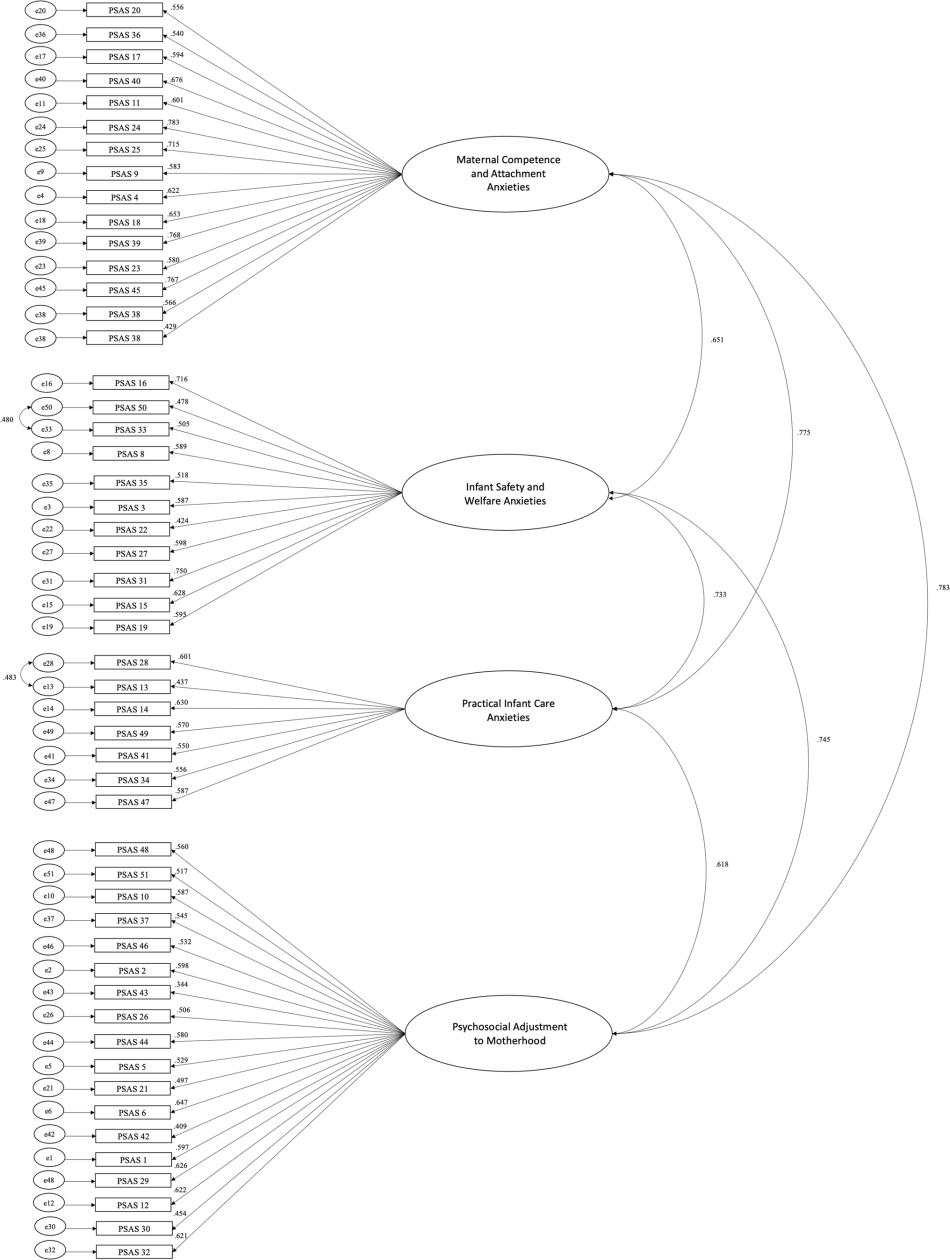
Standardised factor loadings

The initial model was a good fit of the data (CFI = .97, TLI = .97, RMSEA = 0.05, SRMR = 0.06). Modification indices indicated a covariance should be added between two pairs of residuals (MI’s ≥ 10). As a result, the model fit improved (CFI = .98, TLI = .98, RMSEA = 0.04, SRMR = 0.06). All items significantly loaded onto each factor (*p* < .001). The four factors had good-to-excellent reliability (F1: ω = .86; F2: ω = .91; F3: ω = .78; F4: ω = .85). The overall scale demonstrated excellent reliability (McDonald’s ω = .95) (see Fig. 1).

### Associations between postpartum specific anxiety and subjective birth experience

The model found significant associations between higher stress and emotional response to childbirth and higher psychosocial adjustment to motherhood anxieties (B = -1.00 SE = .40, *p* = .012). The model also found significant associations between low quality of care experienced during childbirth and high competence and attachment anxieties (B = 0.84 SE = 0.31, *p* = .007); high infant safety and welfare anxieties (B = 0.61 SE = .26, *p* = .002); and high practical infant care anxieties (B = -1.08 SE = .43, *p* = .001) (see Table 3).

**Table 3:** Total effects of associations between PSAS subscales and subjective birth experience (B(SE) = unstandardised coefficients)

	B (SE), p			
Subjective birth experience	Factor 1	Factor 2	Factor 3	Factor 4
Quality of care experience	.84 (.31), **.007**	.61 (.26), **.002**	-1.08 (.43), **.001**	-.08 (.29), .776
Stress and emotional response	.19 (.04), .648	-.10 (.35), .776	-.63 (.56), .258	-1.00 (.40), **.012**

N.B. Significant values in bold

### Associations between postpartum specific anxiety and objective birth experiences

The model found significant associations between admission to the neonatal care unit and practical infant care anxieties (OR = 0.90; 95% CI = 0.83–0.98, *p* = .01) and asphyxia and infant safety and welfare anxieties (OR = 0.84; 95% CI = 0.73–0.98, *p* = .03) (see Table 4).

**Table 4:** Generalised linear model between PSAS subscales and objective birth experiences

	Odds ratio (CI 95%), p			
Objective birth experience	Factor 1	Factor 2	Factor 3	Factor 4
Caesarean section	1.01 (0.98—1.05), *p* = .45	0.99 (0.95 -1.03), *p* = .58	0.96 (0.90—1.02), *p* = .16	0.98 (0.95—1.01), *p* = .16
Induction	0.99 (0.96—1.02), *p* = .48	1.00 (0.97—1.04), *p* = .87	1.00 (0.95—1.06), *p* = .97	1.00 (0.97—1.03), *p* = .97
Augmentation	1.01 (0.97—1.06), *p* = .57	0.96 (0.91—1.01), *p* = .11	1.02 (0.94—1.11), *p* = .65	0.97 (0.93—1.01), *p* = .10
Neonatal care	1.03 (0.98—1.08), *p* = .24	1.00 (0.94—1.06), *p* = .94	0.90 (0.83 – 0.98), ***p*** **=** **.01**	1.00 (0.95—1.04), *p* = .84
Forceps ventouse	0.99 (0.93—1.06), *p* = .82	0.97 (0.91—1.04), *p* = .44	1.00 (0.90—1.11), *p* = .95	1.02 (0.96—1.07), *p* = .95
Postpartum haemorrhage	0.95 (0.91—1.00), *p* = .07	0.95 (0.90—1.12), *p* = .13	1.02 (0.98—1.07), *p* = .30	1.02 (0.98—1.07), *p* = .30
Breech	1.02 (0.97—1.08), *p* = .42	0.99 (0.93—1.06), *p* = .87	0.95 (0.86—1.04), *p* = .28	0.98 (0.93—1.03), *p* = .46
Postpartum infection	0.99 (0.93—1.06), *p* = .82	0.97 (0.91—1.04), *p* = .44	1.00 (0.90—1.11), *p* = .95	1.02 (0.96—1.07), *p* = .58
Asphyxia	1.01 (0.90—1.14), *p* = .85	0.84 (0.73—0.98), ***p*** = **.03**	1.06 (0.85—1.31), *p* = .63	0.99 (0.89—1.10), *p* = .85
Placenta previa	1.08 (0.95—1.24), *p* = .25	0.89 (0.76—1.04), *p* = .16	0.99 (0.79—1.24), *p* = .94	0.98 (0.88—1.10), *p* = .78
Umbilical cord issues	1.00 (0.93—1.07), *p* = .96	0.95 (0.88—1.04), *p* = .27	1.00 (0.88—1.13), *p* = .96	1.02 (0.95—1.08), *p* = .61
Perineal laceration	0.97 (0.92—1.03), *p* = .33	1.03 (0.96—1.10), *p* = .42	1.06 (0.97—1.17), *p* = .21	1.00 (0.96—1.05), *p* = .88

N.B. Significant values in bold

## Discussion

The current study confirmed the multi-dimensional factor structure of the original 51-item English language PSAS. The four-factor structure mirrors the findings of the initial exploratory factor analyses conducted with the 51-item PSAS in 2016 with the same individual items retained on each factor. The PSAS domains are expressed as: (1) maternal competence and attachment anxieties; (2) infant safety and welfare anxieties; (3) practical infant care anxieties; and (4) psychosocial adjustment to motherhood. The final model fits the data well and internal consistency was excellent, providing further supporting evidence for the reliability and construct validity of the PSAS. Importantly, this study also extends the utility of the PSAS for research across the first postpartum year.

The confirmed structure also reflects more recent validations using the briefer derivatives of the PSAS: the 16-item Research Short Form (PSAS-RSF; Davies et al. 2021), and the 12-item Research Short Form for use in global Crises (PSAS-RSF-C; Silverio et al. 2021). A four-factor structure has also been identified in international validations of the PSAS in France (PSAS-FR; Infante-Gil et al. 2021) and Iran (PSAS-IR; Hasanzadeh et al. 2021) indicating these domains of anxiety hold constant across cultures and countries with differing income levels. Further international validations are underway to confirm this more widely, and measurement invariance analyses are an important next step to determine whether the PSAS is interpreted in the same way across different countries.

The second aim of the study was to model associations between subjective and objective birth experiences and the individual PSAS subscales in the first postpartum year. The findings provide further evidence for the convergent validity of the PSAS, and also support our hypothesis, in that elements of both subjective and objective birth experience were associated with different domains of postpartum anxiety. In terms of subjective birth experience, poorer perceptions of quality of care experienced during birth were associated with higher levels of maternal competence and attachment anxieties, infant safety and welfare anxieties, and practical infant care anxieties. These findings reflect other work which has demonstrated the importance of quality of care during birth for maternal self-efficacy (Bryanton et al. 2008), and alleviating anxiety more generally within the parenting role (Macdorman 2018). Negative stress and emotional responses during birth (feelings of distress and lack of control) were also associated with psychosocial adjustment to motherhood anxieties in the first postpartum year. This indicates that women experiencing feelings of high stress and negative emotion during labour may also experience similar emotional dysregulation during other key adjustments associated with the transition to motherhood, measured in the PSAS as changes in personal appearance, relationships and support, work, finances, and sleep. Taken together, these results resonate with other work which highlights the importance of positive subjective birth experiences for maternal well-being (Andersen et al. 2012; Ayers et al. 2016; Dekel et al. 2017; Grekin and O'Hara, 2014; Simpson and Catling 2016). It is important to note this study was cross-sectional and therefore we were unable to examine history of mental health in the sample. Therefore, those mothers with pre-existing anxiety may have had poorer subjective birth experiences and subsequently higher levels of postnatal anxiety.

Objective birth experience findings demonstrated infant NICU admission was associated with practical infant care anxieties across the first postpartum year. Infant admissions to NICU typically involve alteration to the immediate caregiving role, which has been noted in a recent review as the principal stressor for NICU parents (Govindaswamy et al. 2019). These alterations involve separation from the infant and inability to perform daily care tasks including feeding, bathing, and soothing (Dudek-Shriber 2004) which are all measured by the practical infant care anxiety subscale of the PSAS. Our findings also highlighted an association between infant asphyxia during birth and subsequent infant safety and welfare anxieties. During birth, asphyxia occurs when the infant suffers a combination of oxygen deficiency and reduced blood supply (Azzopardi et al. 2014). Infant asphyxia poses an immediate threat to infant safety and welfare and usually requires urgent medical intervention (Nassef et al. 2013). Concerns around accidental harm and potential death are central to the infant safety and welfare anxiety PSAS subscale. These findings, in combination with those relating to subjective birth experience, suggest specific experiences which occur during birth may elicit a differential anxiety response, i.e. they are related to specific forms of postpartum anxiety occurring during the first postpartum year.

However, this differential anxiety response hypothesis is tentative as it was not supported in the other objective birth experience analyses. Both infant admission to NICU and infant asphyxia are serious and often unanticipated obstetric experiences with serious implications for infant health. Early stress research demonstrated the magnitude of a particular stressor determines the level and duration of the stress response (Horowitz et al. 1980) , which may explain why significant findings were observed for these objective variables and not others examined (e.g. caesarean section; induction of labour). Women’s subjective appraisal of birth has consistently found to be more important in terms of birth satisfaction and mental wellbeing, than objective obstetric birth experience (Baptie 2021). This may also explain why only certain severe obstetric experiences had relationships with specific forms of anxiety, whereas both subscales of subjective birth satisfaction demonstrated significant associations. Further work is needed to test this preliminary hypothesis, but replication of these findings could offer interesting opportunities for targeted intervention of specific anxieties based on specific birth experiences.

Should findings be replicated in other studies, healthcare professionals should be made aware that if mothers experience certain complications or interventions during birth, they may be more prone to experiencing certain forms of anxiety and therefore can be supported appropriately. For example, upon discharge from NICU, mothers may be offered further information on routine infant care tasks, which may alleviate these anxieties. Likewise, for mother of a baby who experienced infant asphyxia, additional reassurance from postnatal care providers should be offered to ameliorate infant safety and welfare anxieties upon discharge. Finally, if women present to primary care services or are noted in universal postnatal care provision, to have particular anxieties, healthcare providers should explore whether these anxieties are a result of an unresolved negative birth experience. Unlike the general anxiety measures currently used in clinical practice, using childbearing specific psychometric tools, such as the PSAS, may therefore facilitate conversations between healthcare providers and women who are presenting with postpartum anxiety symptoms. Future work to develop a modified clinical version of the PSAS is both necessary and imminent.

### Strengths, limitations, and future directions

Our sample size was adequate for the analyses performed, but a common problem in perinatal research conducted with online samples is homogeneity in demographic characteristics (Vignato et al. 2019). As such, the sample lacked diversity and comprised predominately White, married, university-level educated, professional women. Innovative methods of survey recruitment are necessary to achieve the sample sizes necessary for psychometric research while diversifying samples to allow for better generalisability. Further limitations of this study include: multiple objective birth experiences were captured, but analyses were not controlled for multiplicity; and a lack of exploration of how anxiety symptomatology might vary across the first postpartum year. Timing and effect of multiple interventions or complications would be important to explore in future studies. Most psychometric studies use analyses appropriate for interval-level data despite self-report psychological measures being ordinal in nature. In the present study, the use of polychoric correlation matrices which are ordinal level methods of analysis overcomes this problem (Kolenikov and Angeles 2004). Furthermore, the use of McDonald’s omega is statistically more robust than Cronbach’s alpha for the assessment of reliability (Revelle and Zinbarg 2009). Finally, structural equation modelling allowed us to test associations between birth experiences and postpartum anxiety while removing the potential for residual error in the data set. Longitudinal work using the PSAS is now necessary to test associations between birth experience and the PSAS subscales further with measurement of anxieties at different stages of the postnatal period. This design could also incorporate other established antenatal and intrapartum risk factors for postpartum anxiety (e.g. fear of childbirth; birth trauma) to examine whether differences in anxiety response occur postnatally.

## Conclusion

The current study confirmed the four-factor structure of the original 51-item English language PSAS and adds to a growing body of international evidence for the validity and reliability of the tool for assessing maternal and infant focused anxieties. We also provide the first evidence for the convergent validity of the PSAS in relation to subjective birth experiences. Notably, findings suggest specific objective and subjective experiences which occur during birth are related to specific forms of postpartum anxiety—a *differential anxiety response hypothesis* is therefore postulated*.* Further longitudinal studies are needed to examine the relationship between birth experiences and the PSAS subscales further and test this hypothesis.

## Data Availability

The data used in this manuscript form part of a common dataset for a programme of work dedicated to the ongoing translation, adaptation, and validation of the PSAS. Applications for use of the data can be made to the PSAS Chief Investigator (Dr. V. Fallon) on reasonable request. All applications will be ratified by the PSAS Working Group, and any publications resulting from analyses will have to credit the PSAS Working Group and the common dataset.
